# Effects of Combined Exercise Training on Modulating Fine Particulate Matter–Induced Skeletal Muscle Damage in Offspring Gestationally Exposed

**DOI:** 10.1002/jcsm.70047

**Published:** 2025-09-10

**Authors:** Zilin Wang, Wenduo Liu, Hyun‐Jaung Sim, Jeong‐Chae Lee, Sung‐Ho Kook, Sang Hyun Kim

**Affiliations:** ^1^ Department of Sports Science, College of Natural Science Jeonbuk National University Jeonju Republic of Korea; ^2^ Cluster for Craniofacial Development and Regeneration Research, Institute of Oral Biosciences and School of Dentistry Jeonbuk National University Jeonju Republic of Korea; ^3^ Department of Bioactive Material Sciences, Research Center of Bioactive Materials Jeonbuk National University Jeonju Republic of Korea

**Keywords:** combined exercise training, fine particulate matter, mitochondrial function, sex differences, skeletal muscle development

## Abstract

**Background:**

Fine particulate matter has developmental toxicity, and midgestation is an important period for the development of foetal skeletal muscle. The ability of exercise to modulate skeletal muscle damage in mice exposed to PM_2.5_ during gestation remains unclear.

**Methods:**

Pregnant C57BL/6 mice were exposed to 50 μg/m^3^ PM_2.5_ for 2 h on five consecutive days starting at embryonic day 12.5 (E12.5d). Combined exercise (treadmill endurance training and weighted ladder resistance training) was followed for 8 weeks in the 4‐week‐old offspring to verify the regulatory effect of exercise.

**Results:**

Offspring exposed to PM_2.5_ during gestation showed lower body weight (male, −44.3%; female, −44.8%; *p* < 0.001), lower skeletal muscle mass (male: TA fibre size, −42%, *p* < 0.001; TA mass, −37%, *p* < 0.01; gastrocnemius mass, −46.5%, *p* < 0.001; female: TA fibre size, −51.6%, *p* < 0.001; TA mass, −29.8%, *p* < 0.05; gastrocnemius mass, −40.7%, *p* < 0.01) and mitochondrial (size decreased for TEM; male: PGC‐1α, +78.1%, *p* < 0.05; Tfam, +591.3%, *p* < 0.001; FIS‐1, +627%, *p* < 0.001; female: Tfam, +452%, *p* < 0.01; FIS‐1, +345.6%, *p* < 0.01) dysfunction (at 4 weeks old). They also showed catch‐up growth (between 3 and 8 weeks of age; male average weight gain level, +57.9%, *p* < 0.01; female average weight gain level, +66%, *p* < 0.05), although they still showed significant mitochondrial damage and impaired glucose metabolism (at 13 weeks of age; male: mitochondrial damage for TEM; Tfam, −46%, *p* < 0.01; PINK‐1, −33.8%, *p* < 0.05; Parkin, −62%, *p* < 0.01; PFK‐1, −17%, *p* < 0.05; female: mitochondrial damage for TEM; PFK‐1, −28.7%, *p* < 0.01). Combined exercise was unable to regulate the skeletal muscle system disorder that occurred in male offspring exposed to PM_2.5_ during pregnancy. However, it activated the mitophagy (PINK‐1, +94.6%, *p* < 0.05; Parkin, +90.2%, *p* < 0.001) in female offspring exposed to PM_2.5_ during pregnancy, thereby improving mitochondrial damage.

**Conclusions:**

Combined exercise had bidirectional, sex‐specific effects: Male offspring exhibited reduced responsiveness to exercise, with persistent mitochondrial damage, whereas female offspring showed improved mitochondrial health through increased mitophagy flux.

## 1 Introduction

Fine particulate matter (PM_2.5_) is a prevalent air pollutant linked to numerous adverse health outcomes [1], including acute lung injury [2], cardiovascular diseases [3], haematopoietic stem cell ageing [4] and skeletal muscle mitochondrial damage [5]. While the effects of direct PM_2.5_ exposure are well documented, the consequences of prenatal exposure remain poorly understood [6]. Gestational exposure to air pollutants has been associated with an increased risk of preterm birth, low birth weight and intrauterine growth restriction, potentially driven by oxidative stress, inflammation and endothelial dysfunction [7]. However, the long‐term effects of prenatal PM_2.5_ exposure on skeletal muscle development and function in offspring are not fully explored.

Gestational PM_2.5_ exposure may lead to structural and functional deficits in skeletal muscle, including growth retardation, reduced muscle mass, smaller muscle fibre size and impaired performance such as diminished grip strength and endurance capacity [8]. At the cellular level, PM_2.5_ exposure disrupts mitochondrial function, evident in imbalances in mitochondrial fusion and fission processes, reduced mitochondrial size and increased oxidative stress [9]. Moreover, PM_2.5_ exposure impairs glucose metabolism in skeletal muscle, as reflected in the decreased expression of key regulators like glucose transporter type 4 (GLUT‐4), which are essential for glucose uptake and glycolysis [5]. These deficits are particularly concerning as skeletal muscle plays a vital role in maintaining metabolic health, energy expenditure and overall physical performance.

Exercise is known to promote skeletal muscle health through several key mechanisms. It upregulates mitochondrial biogenesis, with factors such as peroxisome proliferator‐activated receptor gamma coactivator 1‐alpha (PGC‐1α) playing a crucial role in mitochondrial growth and energy production [10]. Additionally, exercise activates protein synthesis pathways like mTOR signalling, contributing to muscle hypertrophy [11]. It also suppresses muscle atrophy by downregulating catabolic factors such as muscle ring‐finger protein‐1 (MuRF‐1) and muscle atrophy F‐box (MAFbx), which are involved in muscle protein degradation [12]. These effects together enhance mitochondrial dynamics, antioxidant defences and glucose metabolism, underscoring the potential of exercise to counteract the skeletal muscle damage induced by PM_2.5_ exposure.

This study investigates the long‐term effects of gestational PM_2.5_ exposure on offspring skeletal muscle and examines whether combined exercise training can mitigate these deficits. By focusing on mitochondrial dynamics, muscle regeneration and glucose metabolism, this research aims to identify therapeutic strategies to alleviate the developmental impacts of prenatal environmental exposures.

## 2 Methods

### 2.1 Animals

C57BL/6 mice were purchased from Damul Science (Korea) and time‐mated to maximize birth cohorts. The sex of neonatal mice was not determined, and both male and female neonates were included in the experiments in specific pathogen‐free conditions. The room in which the mice were housed was maintained at a controlled temperature (18°C–22°C) and humidity (40%–60%) on a 12‐h light–dark cycle. Water and feed were provided ad libitum during the breeding process. All experimental procedures were conducted in accordance with the guidelines and regulations set by the Institutional Animal Care and Use Committee of Jeonbuk National University (IACUC approval no. CBNU‐2023‐114).

### 2.2 Experimental Design

**FIGURE 1:**
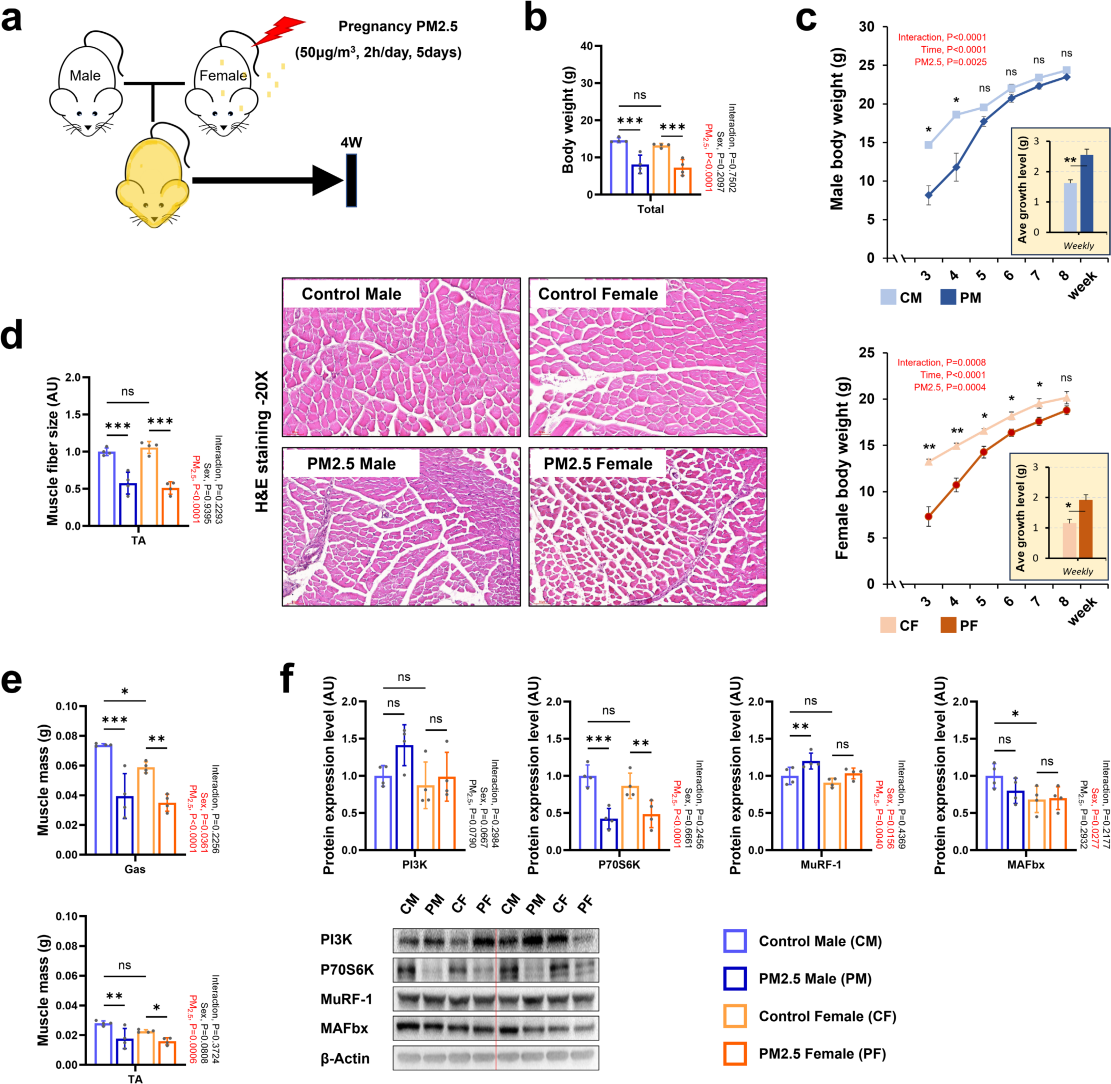
The effect of PM_2.5_ exposure during pregnancy on offspring development. (a) Schematic experimental design. (b) Body weight in 4 weeks of age. (c) Weight changes and growth rate in offspring. (d) Muscle fibre size and representative H&E staining images of tibialis anterior in 4 weeks of age. (e) Tibialis anterior and gastrocnemius masses in 4 weeks of age. (f) Representative western blot images and expression levels of PI3K, P70S6K, MuRF‐1 and MAFbx in the gastrocnemius muscle at 4 weeks of age. Every group *n* = 4. Data were presented as mean ± SD. Data were analysed by two‐way ANOVA or two‐sided unpaired Student's *t*‐tests (*p < 0.05; **p < 0.01; ***p < 0.001; ns, not significant, *p* > 0.05).

This study is divided into two main parts. We first verified the skeletal muscle developmental effects of PM_2.5_ exposure during pregnancy in offspring at 4 weeks of age (Figure 1a). After confirming the negative impact of PM_2.5_ on the skeletal muscles, we verify the regulatory effect of combined exercise during the growth period of offspring exposed to PM_2.5_ during pregnancy (Figures 3a and 6a). The period of PM_2.5_ exposure during pregnancy in the experiment all started on embryonic day 12.5. The combined exercise programme performed in the experiment is shown in Figure S2. After a week of acclimatization (three times each on the treadmill and climbing ladder), the 4‐week‐old offspring began an 8‐week period of combined exercise. Training was performed five times per week: Monday, Wednesday and Friday were resistance training days (the warm‐up was one‐time unloaded ladder climbing, followed by 5–10 sets of ladder climbing with weights). For the first 2 weeks, the tail of the mouse was loaded with 10% of its body weight, 20% from weeks 3 to 6 and 40% from weeks 7 to 8. When the mice stopped crawling during the exercise, the researchers stimulated their tails and hind limbs with a sponge to complete the crawling task. If a mouse falls during the ladder climb, it is reset to the bottom of the ladder, allowed to rest for 10 s, and then restarts. Climbs that do not reach the top will not be counted towards the exercise group. Tuesday and Thursday were endurance training days on a treadmill (5° slope). The warm‐up was performed at a speed of 8 m/min for 5 min, followed by running at a speed of 10 m/min for 20 min. Cool down was performed for 5 min at a speed of 8 m/min. The main training speed is increased once every 2 weeks, with each increase equal to 5% of the maximum running speed. When the mice could no longer run and remained on an electric shock grid where no current was applied, they were stimulated to continue running by stimulating their tails and hind limbs with a sponge at low‐to‐medium intensity. The intensity of endurance exercise and the load of resistance exercise gradually increased with the exercise cycle (see Figure S2 for specific intensities). Sample collection for analysis of the regulatory effect of exercise was performed 18 h after the last exercise for all mice.

### 2.3 Chemical Composition of PM_2.5_

Organic components and inorganic salts used to prepare artificial PM_2.5_ are listed in Table S1. Eight organic compounds with carboxylic acid, polyol, sugar, aromatic and/or amino acid functional groups were investigated. Ammonium sulfate and ammonium nitrate were used as the model inorganic salts because of their abundance in air (Reference S1–S3). The 10 compounds in Table S1 were mixed at an organic‐to‐inorganic dry mass ratio of 1:1 to mimic the chemical complexity of atmospheric aerosols. Compounds were purchased from Sigma‐Aldrich (purity ≥ 98%) and were used without further purification. The 10 components were dissolved in purified water.

### 2.4 Atmospheric Simulation Chamber (ASC) System

The ASC system is a whole‐body exposure device designed to replicate the inhalation of PM_2.5_ in the atmosphere while maintaining a consistent average concentration. PM solution was formulated by mixing 10 organic and inorganic compounds, namely, oxalic acid, malonic acid, glutaric acid, sucrose, 2,5‐dihydroxybenzoic acid, glycine, ammonium sulfate, ammonium nitrate, acetate and glycerol, into distilled water. PM was aerosolized using a nebulizer (TQ‐50‐C0.5; Meinhard, USA) and, after passing through a polypropylene melt‐blown filter, particles larger than 2.5 μm were sieved out. The concentration of PM_2.5_ within the chamber was continuously monitored in real time using a particle counter (BT‐610; Met One, USA), with the predetermined concentration automatically maintained using a flow controller programme. To expose female mice to PM_2.5_ at embryonic days 12.5–16.5, mice were exposed to PM_2.5_ for 2 h per day, five times a week, at a concentration of 51.8 ± 9.4 μg/m^3^ (Table S2). The humidity level in the chamber was maintained at 55%–60% and the temperature in the range of 23°C–25°C. The exposure dose and duration were selected based on our previous study (Reference S4), where a concentration of 50 μg/m^3^ was used to expose conscious pregnant mice over 5 days, resulting in induced lung inflammation and oxidation in the offspring.

### 2.5 Grip Strength Test

The peak grip strengths of the forelimbs and limbs of mice were evaluated using a grip strength meter (Ugo Basile, Cat. No. 47200, Italy). During the test, the mouse's forelimbs/limb grasp the test bar/net, and the tension on the mouse's tail is gradually increased until the mouse is detached from the bar/net to obtain the maximum peak of the grip force. Each test was performed five times per mouse to ensure reliable maximal values (Reference S5).

### 2.6 Endurance Exercise Capacity (EEC) Test

A progressive EEC test was conducted to assess the impact of endurance training. The EEC test was performed on a treadmill with a fixed slope of 15°. Initially, the treadmill speed was set at 10 m/min for the first 5 min, and subsequently, the speed was increased by 2 m/min every minute. During the test, if a mouse was unable to continue running despite sponge stimulation and remained on the electric shock grid for more than 3 s, the test was stopped. The total running time, vertical distance and work were measured or calculated using methodologies described in previous studies [5].

### 2.7 Tissue Mass Analysis

At the time of skeletal muscle collection, epididymal (Epi) or ovarian (Ova), and retroperitoneal (Ret) adipose tissue mass was measured using an electronic balance (Sartorius Lab Instruments GmbH & Co. KG, 37070 Goettingen, Germany).

### 2.8 Western Blot Analysis

Gastrocnemius (Gas) muscle extracts were prepared, and western blotting was performed as described previously [5]. The antibodies used are as follows: PGC‐1α (GeneTex, GTX37356, USA); mitochondrial transcription factor A (Tfam; SCBT, sc‐166 965, USA); oxidative phosphorylation (OXPHOS, Abcam, ab110413, USA); mitofusin 1 (MFN‐1; SCBT, sc‐166 644, USA); mitofusin 2 (MFN2; SCBT, sc‐515 647, USA); mitochondria fission 1 protein (FIS‐1; SCBT, sc‐376 447, USA); dynamin‐related protein 1 (DRP‐1; SCBT, sc‐32 898, USA); PTEN‐induced kinase 1 (PINK‐1; SCBT, sc‐517 353, USA); Parkin (SCBT, sc‐32 282, USA); phosphoinositide 3‐kinases (PI3K; SCBT, sc‐1637, USA); p70S6 kinase (p70S6k; SCBT, sc‐8418, USA); MuRF‐1 (SCBT, sc‐398 608, USA); MAFbx (SCBT, sc‐166 806, USA); GLUT‐4 (SCBT, sc‐53 566, USA); hexokinase 2 (HK2, SCBT, sc‐374 091, USA); phosphofructokinase‐1 (PFK‐1; SCBT, sc‐166 722, USA); lactate dehydrogenase (LDH, SCBT, sc‐133 123, USA); β‐actin (Invitrogen, MA1–140, USA); mouse anti‐goat (SCBT, sc‐2354, USA); mouse anti‐rabbit (SCBT, sc‐2357, USA).

### 2.9 Transmission Electron Microscopy (TEM)

TEM was used to examine mitochondrial structure in the soleus muscle. For each group, we analysed three biological replicates and randomly selected six to eight TEM fields per sample at the same magnification. The muscle was fixed with 2.5% glutaraldehyde and 4% formaldehyde in 0.1‐M phosphate buffer (pH 7.4) for 2 h, followed by post‐fixation with 1% osmium tetroxide for 2 h. After dehydration in a graded ethanol series, the muscle was embedded in Epon‐812 resin. Thin sections (~80 nm) were cut using a NOVA ultramicrotome (LKB, Vienna, Austria) and mounted on a 100‐mesh grid. Sections were stained with uranyl acetate and lead citrate and examined with an electron microscope (H7650, 80 kV, Hitachi, Japan). TEM analysis was performed using a JEM‐2010 microscope (JEOL) at the Center for University‐Wide Research Facilities (CURF) at Jeonbuk National University.

TEM images were independently reviewed by two examiners who were unaware of the sample information. The examination aimed to assess the following four indicators to verify the level of mitochondrial damage: (1) loss or disruption of cristae structure, (2) swelling of the mitochondrial matrix, (3) compromised outer membrane integrity and (4) the presence of small vacuoles within the mitochondria. These criteria were consistently applied to all samples.

### 2.10 Haematoxylin and Eosin (H&E) Staining

Histologic analysis of tibialis anterior (TA) muscle samples was performed according to a previously developed protocol [4]. Tissues were fixed using formalin at 4°C. After dehydration with ethanol, tissue samples were clarified, infiltrated and paraffin‐embedded with xylene. Embedded tissues were sectioned and stained with H&E on glass slides. The size of muscle fibres was determined using a Motic Easy Scan One slide scanner (Meyer Instruments Inc., USA) and further quantified using ImageJ software (version 1.51, NIH, USA).

### 2.11 Statistics Analysis

All data are presented as the mean ± standard deviation (SD) and were analysed using GraphPad Software (Prism 10, MA, USA). Differences between two groups were analysed by unpaired Student's *t* test. Two‐way ANOVA followed by Fisher's LSD post hoc test was used for multiple comparisons among groups. A value of *p* < 0.05 was considered statistically significant.

## 3 Results

### 3.1 Effect of Gestational PM_2.5_ Exposure on Skeletal Muscle Development at 4 Weeks of Age

Gestational exposure to fine particulate matter adversely affects the foetus, and its effects are also seen in offspring after birth [13]. Midgestation of mouse embryos is an important period for the development of foetal skeletal muscle through muscle precursor cell–based differentiation and fusion [14]. To investigate how gestational exposure to PM_2.5_ (diameter less than 2.5 μm) affects skeletal muscle development during embryonic development, pregnant mice were exposed to 50 μg/m^3^ PM_2.5_ for 2 h on five consecutive days starting at embryonic day 12.5 (E12.5d), and offspring (4 weeks old) born from exposed pregnant dams were studied (Figure 1a). Given that biological defensive systems respond to stressors differentially depending on sex [15], we analysed skeletal muscle systems in gestationally exposed male and female offspring. Gestational exposure to fine PM_2.5_ caused lower body weights in mice (Figure 1b). Body weight of gestationally PM_2.5_‐exposed males was noticeably reduced at 3 and 4 weeks after birth but recovered to a comparable level as control mice starting at 5 weeks. Female gestationally PM_2.5_‐exposed mice showed a significant decline in body weight that continued up to 7 weeks compared with control offspring (Figure 1c). Stunted development of skeletal muscle was observed in gestationally PM_2.5_‐exposed offspring regardless of sex. Compared with 4‐week‐old offspring born to control dams, those born to PM_2.5_‐exposed dams had measurable decreases in muscle fibre size, as evidenced by H&E stained images of the TA muscle (Figure 1d). This was supported by significant decreases in Gas muscle mass, TA muscle mass and muscle fibre size (Figure 1d,e) in gestationally PM_2.5_‐exposed male and female mice. Furthermore, a significant decrease in the level of p70S6k, one of the skeletal muscle hypertrophic markers [16], and an increase in the level of MuRF‐1, a skeletal muscle atrophy‐related factor, was observed in both sexes of gestationally PM_2.5_‐exposed mice (Figure 1f). The lower skeletal muscle mass of gestationally PM_2.5_‐exposed male and female mice was reflected in the EEC test and grip strength test results (Figure S1a,b). Similar to skeletal muscle mass, Epi or Ova and Ret fat mass statistically decreased in both gestationally PM_2.5_‐exposed male and female mice (Figures 1e and S1c).

**FIGURE 2:**
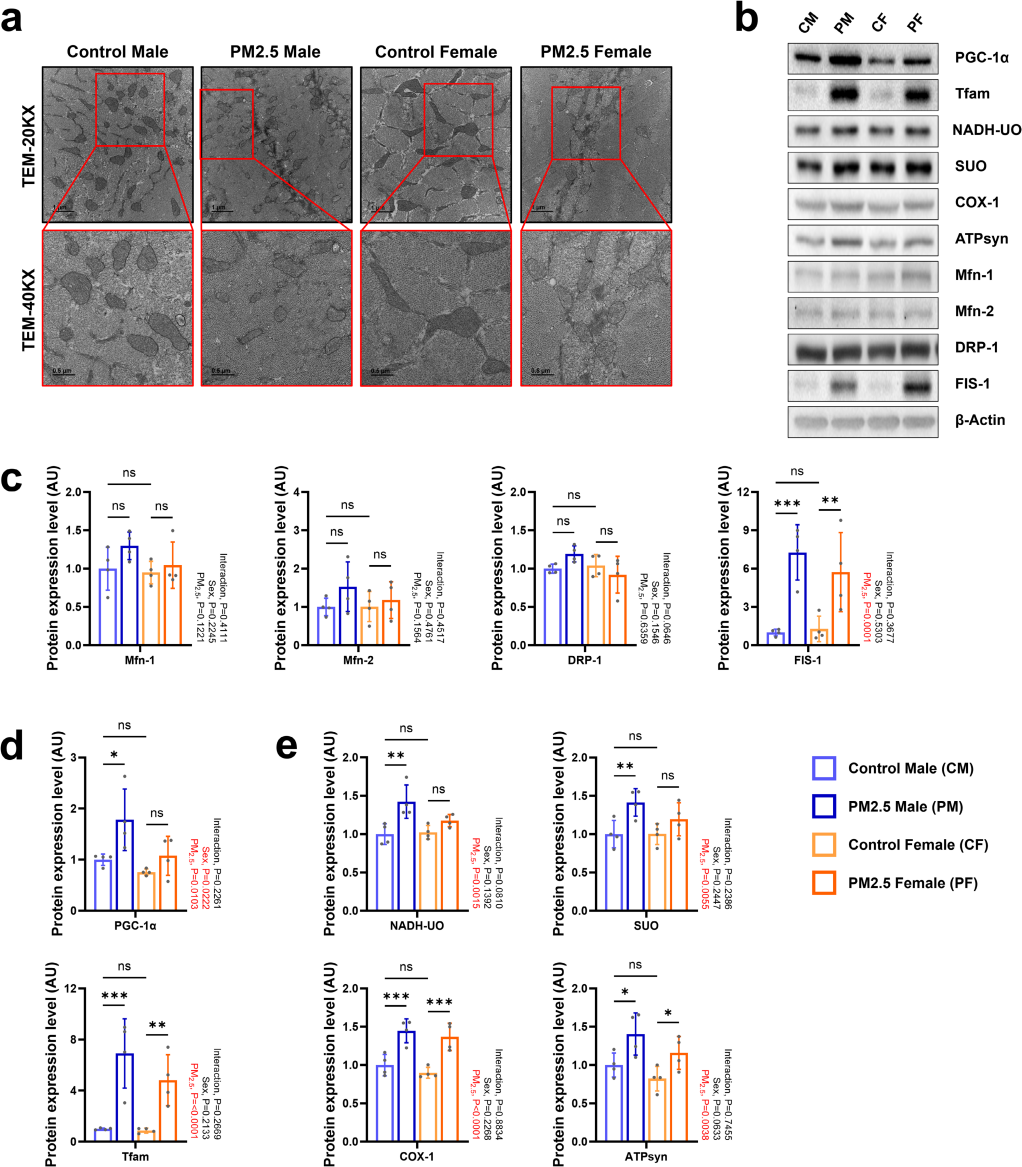
Effect of gestational PM_2.5_ exposure on skeletal muscle mitochondria of offspring 4 weeks of age. (a) Representative images of transmission electron microscopy image of the soleus muscle. The images in the red boxes are enlarged representative images of each sample. (b) Representative western blot images of PGC‐1α, Tfam, NADH‐UO, SUO, COX‐1, ATPsyn, Mfn‐1, Mfn‐2, DRP‐1, FIS‐1 and β‐actin in the gastrocnemius muscle. (c) Expression levels of Mfn‐1, Mfn‐2, DRP‐1 and FIS‐1 in the gastrocnemius muscle. (d) Expression levels of PGC‐1α and Tfam in the gastrocnemius muscle. (e) Expression levels of NADH‐UO, SUO, COX‐1 and ATPsyn in the gastrocnemius muscle. Every group *n* = 4. Data were presented as mean ± SD. Data were analysed by two‐way ANOVA (*p < 0.05; **p < 0.01; ***p < 0.001; ns, not significant, *p* > 0.05).

Because mitochondrial function is closely associated with the modulation of skeletal muscle mass and function [17], we initially assessed morphological alterations in mitochondria from the soleus muscle using TEM. Mitochondrial size was reduced in both sexes of gestationally PM_2.5_‐exposed mice compared with corresponding control mice (Figure 2a). This was likely due to the significant increase in expression of FIS‐1, a mitochondrial fission–associated protein, in both sexes of gestationally exposed mice (Figure 2b,c). No equivalent increases in the levels of mitochondrial fusion–associated proteins such as Mfn‐1 and Mfn‐2, which are mitochondrial fission–associated proteins, were observed, indicating an imbalance in mitochondrial fusion/fission (Figure 2b,c). As a compensatory mechanism, gestationally PM_2.5_‐exposed mice displayed an overall increase in levels of the mitochondrial biogenesis–associated factors PGC‐1α and Tfam (Figure 2b,d) and mitochondrial oxidative phosphorylation of electron transport chain (Oxidative phosphorylation) enzymes such as NADH‐UO, SUO, COX‐1 and ATPsyn (Figure 2b,e) compared with control offspring. Although a compensatory mechanism for the maintenance of mitochondrial homeostasis was observed in gestationally PM_2.5_‐exposed mice, a bias towards mitochondrial fission can contribute to mitochondrial dysfunction [18]. Overall, these results suggest that gestational exposure of mice to atmospherically relevant PM_2.5_ causes developmental abnormalities of skeletal muscle and mitochondrial fission–biased impairment in 4‐week‐old mice.

### 3.2 Effects of Time and Combined Exercise After Gestational PM_2.5_ Exposure on the Restoration of Impaired Skeletal Muscle Function in Male Mice

**FIGURE 3:**
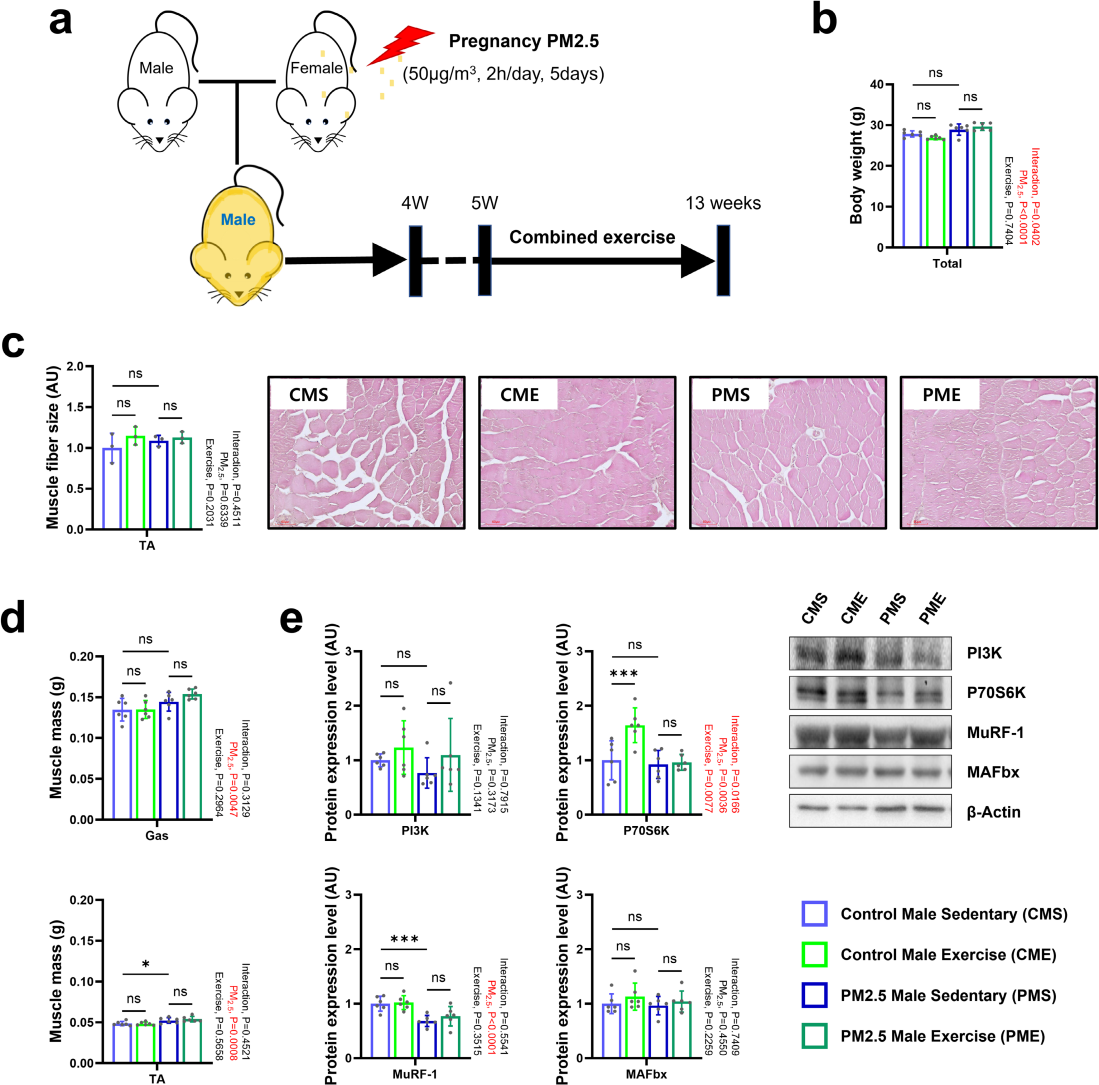
Effect of combined exercise after gestational PM_2.5_ exposure on the body weight and skeletal muscle morphology of male offspring. (a) Schematic experimental design. (b) Body weight (*n* = 6). (c) Muscle fibre size and representative H&E staining images of tibialis anterior (*n* = 3). (d) Tibialis anterior and gastrocnemius masses (*n* = 6). (e) Representative western blot images and expression levels of PI3K, P70S6K, MuRF‐1 and MAFbx in the gastrocnemius muscle (*n* = 6). Data are presented as mean ± SD. Data were analysed by two‐way ANOVA (*p < 0.05; **p < 0.01; ***p < 0.001; ns, not significant, *p* > 0.05).

**FIGURE 4:**
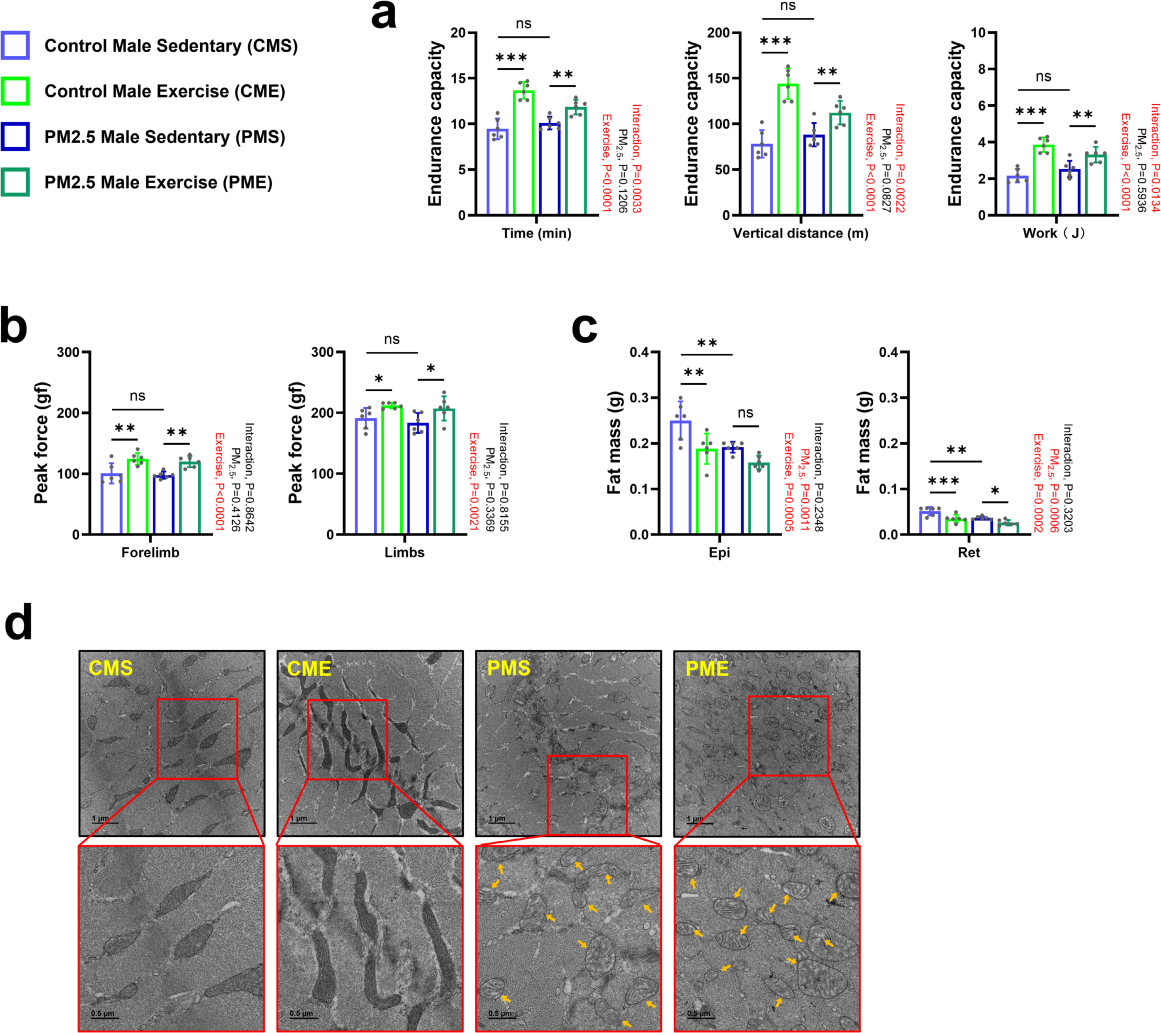
Effect of combined exercise after gestational PM_2.5_ exposure on the exercise capacity, fat mass and mitochondrial damage level of male offspring. (a) Endurance capacity (*n* = 6). (b) Grip strength test (*n* = 6). (c) Epididymal and retroperitoneal fat pad masses (*n* = 6). (d) Representative transmission electron microscopy images of the soleus muscle (*n* = 3). The images in the red boxes are enlarged representative images of each sample. The areas marked with yellow arrows are typical points of mitochondrial damage in the sample. Data are presented as mean ± SD. Data were analysed by two‐way ANOVA (*p < 0.05; **p < 0.01; ***p < 0.001; ns, not significant, *p* > 0.05).

Exercise training promotes skeletal muscle mass recovery and mitochondrial biogenesis after injury [19]. To investigate the effects of long‐term exercise on skeletal muscle and mitochondria impaired by gestational exposure to PM_2.5_, 5‐week‐old offspring were subjected to combined exercise training involving treadmill running (an aerobic exercise) and ladder climbing (a resistance training) for eight consecutive weeks (Figure 3a and Figure S2). Compared with each group, there were no changes in body weight at 13 weeks old (Figure 3b). However, the results of the two‐way ANOVA showed that gestational PM_2.5_ exposure during pregnancy still had an independent effect on offspring body weight and that the interaction between PM_2.5_ and exercise also significantly affected body weight changes at 13 weeks. Moreover, there was no significant difference in skeletal muscle fibre size between the groups (Figure 3c). The decline in mass of the Gas and TA muscles in 4‐week‐old gestationally PM_2.5_‐exposed mice was restored to baseline at 13 weeks old (Figure 3d). Moreover, mice exposed to PM_2.5_ during pregnancy showed an independent effect of PM_2.5_ exposure at the skeletal muscle level at 13 weeks of age, showing higher muscle mass than the control groups. This indicates that the gestational PM_2.5_‐induced impairment in skeletal muscle fibres self‐ameliorates with time. Interestingly, gestationally PM_2.5_‐exposed male sedentary (PMS) offspring showed decreased expression of the skeletal muscle E3 ubiquitination ligase MuRF‐1 after 4 weeks (Figure 3e). However, the positive effect of combined exercise was evident in control male combined exercise (CME) offspring, who showed enhanced expression of the muscle synthesis–related protein, P70S6K; this was not observed in gestationally PM_2.5_‐exposed male mice that exercised (PME) offspring (Figure 3e). As shown in Figure 4a,b, the results of EEC and grip strength tests were comparable between control male sedentary (CMS) and PMS offspring. Combined exercise had a positive effect on the results of both tests in CME and PME offspring compared with CMS and PMS offspring, respectively. The decrease in mass of Epi and Ret fat in gestationally PM_2.5_‐exposed mice remained until 13 weeks of age. Combined exercise caused a reduction in fat mass in both control and gestationally PM_2.5_‐exposed mice (Figure 4c).

**FIGURE 5:**
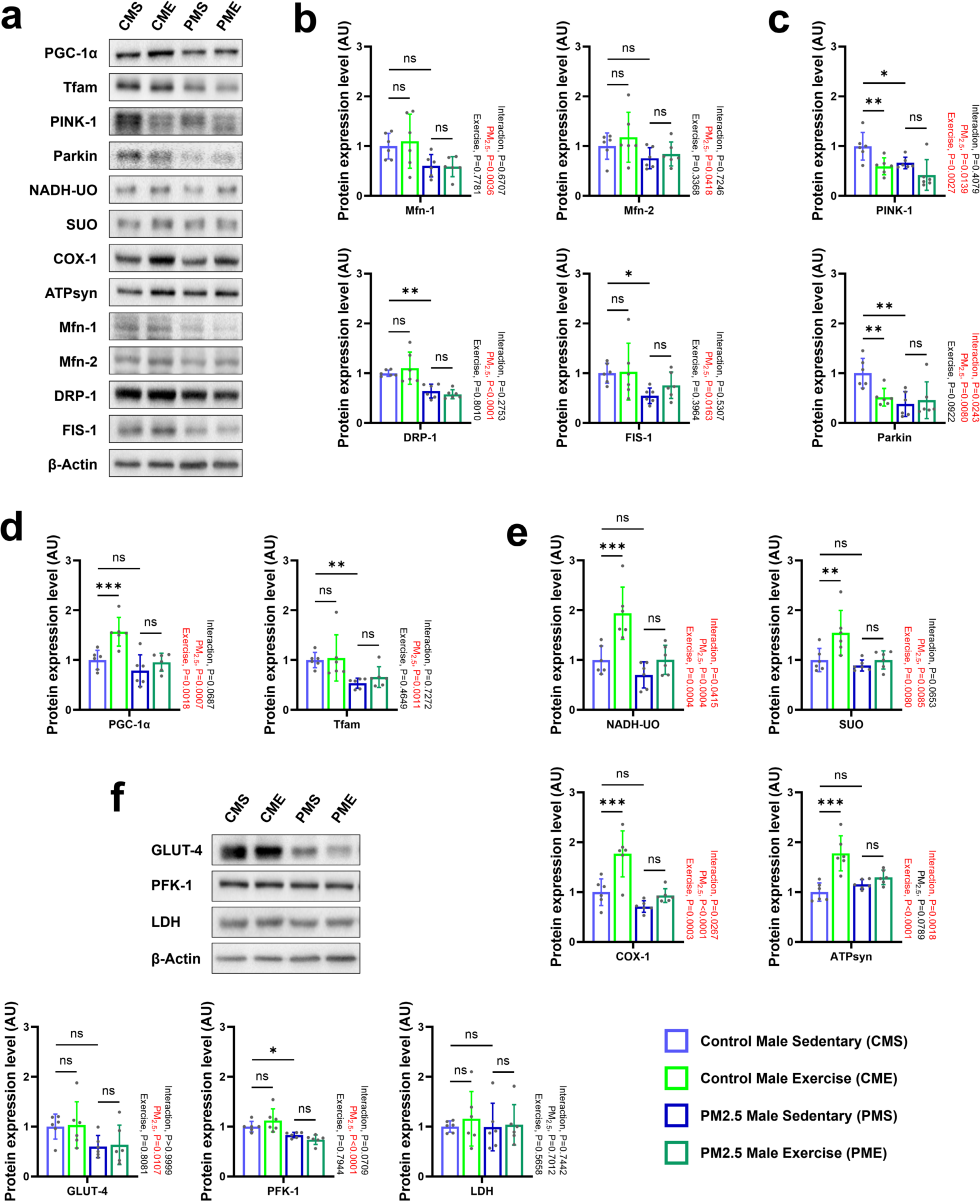
Effect of combined exercise after gestational PM_2.5_ exposure on the skeletal muscle mitochondria and glucose metabolism of male offspring. (a) Representative western blot images of PGC‐1α, Tfam, PINK‐1, Parkin, NADH‐UO, SUO, COX‐1, ATPsyn, Mfn‐1, Mfn‐2, DRP‐1, FIS‐1 and β‐actin in the gastrocnemius muscle. (b) Expression levels of Mfn‐1, Mfn‐2, DRP‐1 and FIS‐1 in the gastrocnemius muscle. (c) Expression levels of PINK‐1 and Parkin in the gastrocnemius muscle. (d) Expression levels of PGC‐1α and Tfam in the gastrocnemius muscle. (e) Expression levels of NADH‐UO, SUO, COX‐1 and ATPsyn in the gastrocnemius muscle. (f) Representative western blot images and expression levels of GLUT‐4, PFK‐1 and LDH in the gastrocnemius muscle. Every group *n* = 6. Data were presented as mean ± SD. Data were analysed by two‐way ANOVA (*p < 0.05; **p < 0.01; ***p < 0.001; ns, not significant, *p* > 0.05).

Different from the recovery of PM_2.5_‐impaired muscle mass with time, the mitochondria in the soleus muscles of PMS offspring remained in a damaged state compared with those of CMS offspring. Despite combined exercise, the mitochondrial damage seen in PMS offspring did not recover appreciably, as evidenced by TEM analyses of PME offspring (Figure 4d). This was supported by the significantly reduced levels in mitochondrial fusion (Mfn‐1) and fission (DRP‐1 and FIS‐1) proteins (Figure 5a,b), and mitophagy (which plays a central role in the repairment and/or formation of new muscle fibres through muscle regeneration and myogenesis by removing damaged mitochondria related proteins [20] PINK‐1 and Parkin; Figure 5a,c) in PMS offspring compared with CMS offspring and the lack of a positive effect of combined exercise on levels of these proteins. Compared with CMS offspring, CME offspring showed no changes in the expression of mitochondrial fusion and fission proteins but did have significantly lower levels of PINK‐1 and Parkin (Figure 5a,c). Of note, a mitochondrial biosynthesis–related factor PGC‐1α (Figure 5a,d) and mitochondrial electron transport chain enzymes NADH‐UO, SUO, COX‐1 and ATPsyn (Figure 5a,e) in CME offspring, but not PME offspring, were activated by combined exercise. Normal skeletal muscle glucose metabolism is essential for the recovery of damaged mitochondria [21]. Compared with CMS offspring, PMS offspring expressed significantly lower levels of muscle glucose metabolism–related proteins such as GLUT‐4 (total expression level of GLUT‐4) and PFK‐1 (Figure 5f). Taken together, these results illustrate that gestational PM_2.5_ impairment of the skeletal muscle system persists in male offspring over time due to disruption of mitochondrial modulation and glucose metabolism; these deficits are not ameliorated by combined exercise.

### 3.3 Effects of Time and Combined Exercise After Gestational PM_2.5_ Exposure on the Restoration of the Impaired Skeletal Muscle in Female Offspring

**FIGURE 6:**
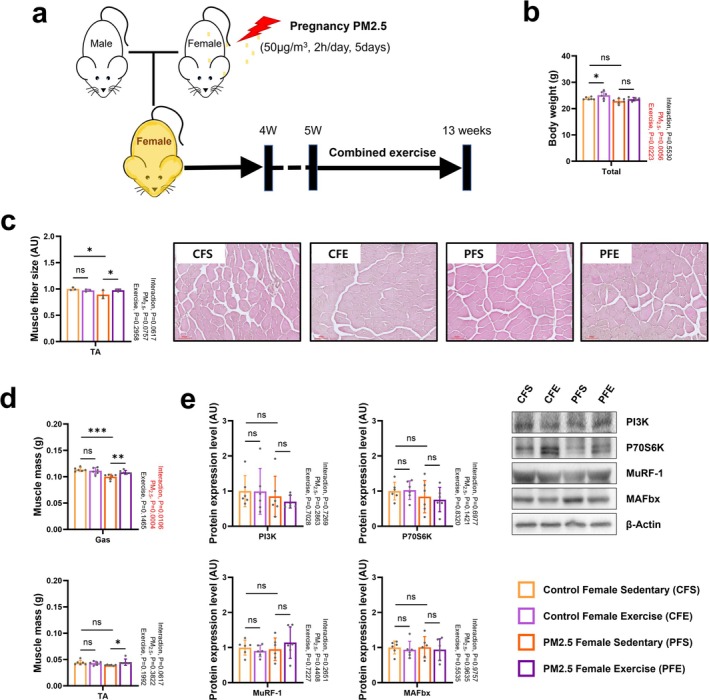
Effect of combined exercise after gestational PM_2.5_ exposure on the body weight and skeletal muscle morphology of female offspring. (a) Schematic experimental design. (b) Body weight (*n* = 6). (c) Muscle fibre size and representative H&E staining images of tibialis anterior (*n* = 3). (d) Tibialis anterior and gastrocnemius masses (*n* = 6). (e) Representative western blot images and expression levels of PI3K, P70S6K, MuRF‐1 and MAFbx in the gastrocnemius muscle (*n* = 6). Data are presented as mean ± SD. Data were analysed by two‐way ANOVA (*p < 0.05; **p < 0.01; ***p < 0.001; ns, not significant, *p* > 0.05).

**FIGURE 7:**
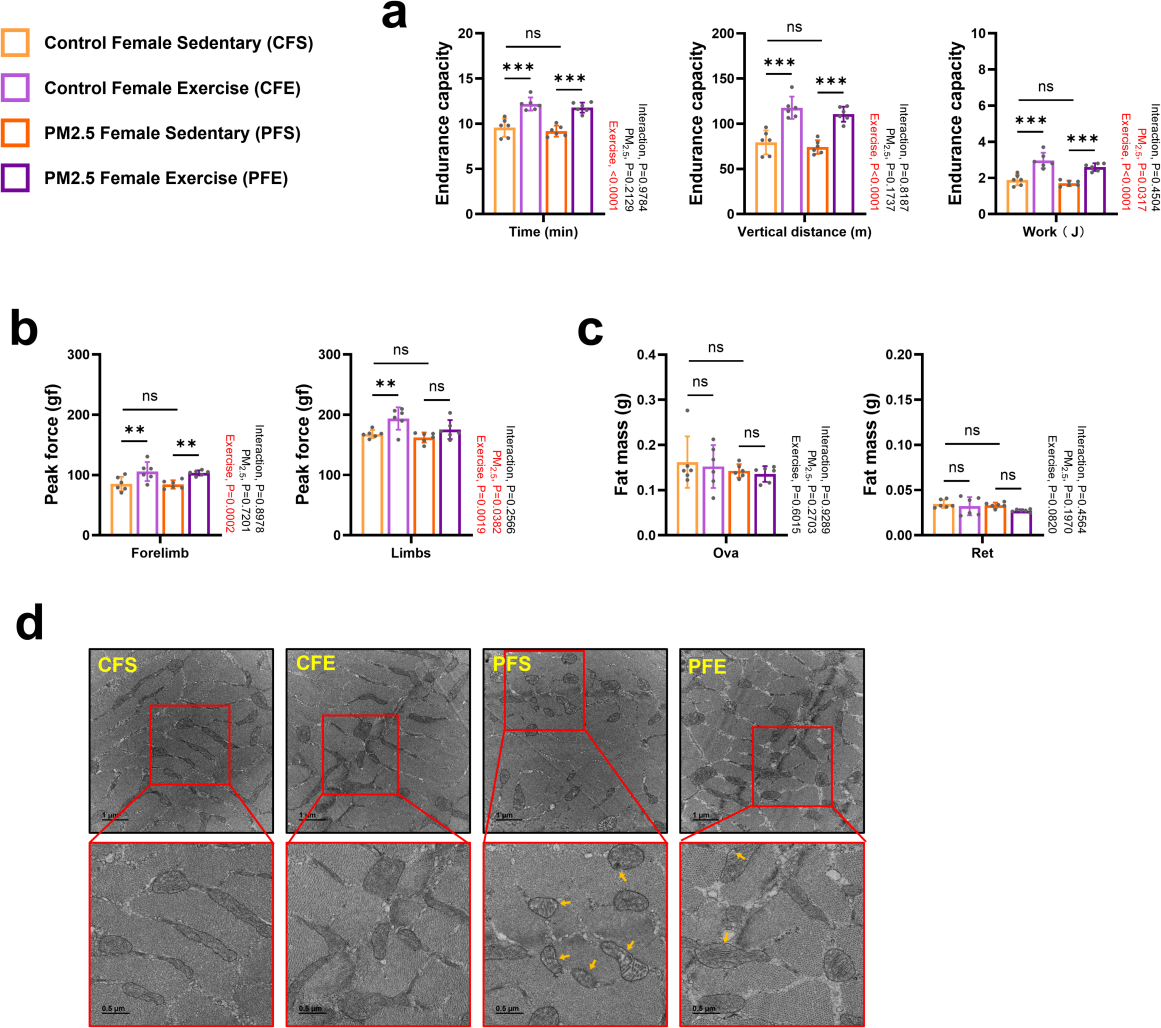
Effect of combined exercise after gestational PM_2.5_ exposure on the exercise capacity, fat mass and mitochondrial damage level of female offspring. (a) Endurance capacity (*n* = 6). (b) Grip strength test (*n* = 6). (c) Ovarian and retroperitoneal fat pad masses (*n* = 6). (d) Representative transmission electron microscopy images of the soleus muscle (*n* = 3). The images in the red boxes are enlarged representative images of each sample. The areas marked with yellow arrows are typical points of mitochondrial damage in the sample. Data are presented as mean ± SD. Data were analysed by two‐way ANOVA (*p < 0.05; **p < 0.01; ***p < 0.001; ns, not significant, *p* > 0.05).

Because the stem cell–based regeneration capacity of tissues differs according to sex [22], we next investigated the restoration of gestationally PM_2.5_‐damaged skeletal muscles in female mice after combined exercise. There was still some difference in body weight between 13‐week‐old control female sedentary (CFS) and gestationally PM_2.5_‐exposed female sedentary (PFS) mice (Figures 6a and S2). Combined exercise did not result in a change in the body weight of either PFS offspring, but CFS offspring body weight was increased with combined exercise (Figure 6b). The PFS offspring still showed some differences in muscle fibre size, but they recovered significantly with combined exercise (Figure 6c). However, different from PMS offspring, PFS offspring still showed a significant reduction in Gas muscle masses compared with CFS offspring (Figure 6d). Of note, combined exercise appeared to be effective at increasing the muscle weight of PFS offspring (Figure 6d). However, combined exercise did not affect the expression levels of proteins associated with pathways related to skeletal muscle protein synthesis and ubiquitination (Figure 6e). Results of the EEC test and grip strength test were comparable between CFS and PFS offspring, both of which showed improved results after combined exercise (Figure 7a,b). Different from PMS offspring, PFS offspring had a similar Ova and Ret fat masses to CFS offspring (Figure 7c). The combined exercise also did not significantly affect the body fat content of the offspring (Figure 7c).

**FIGURE 8:**
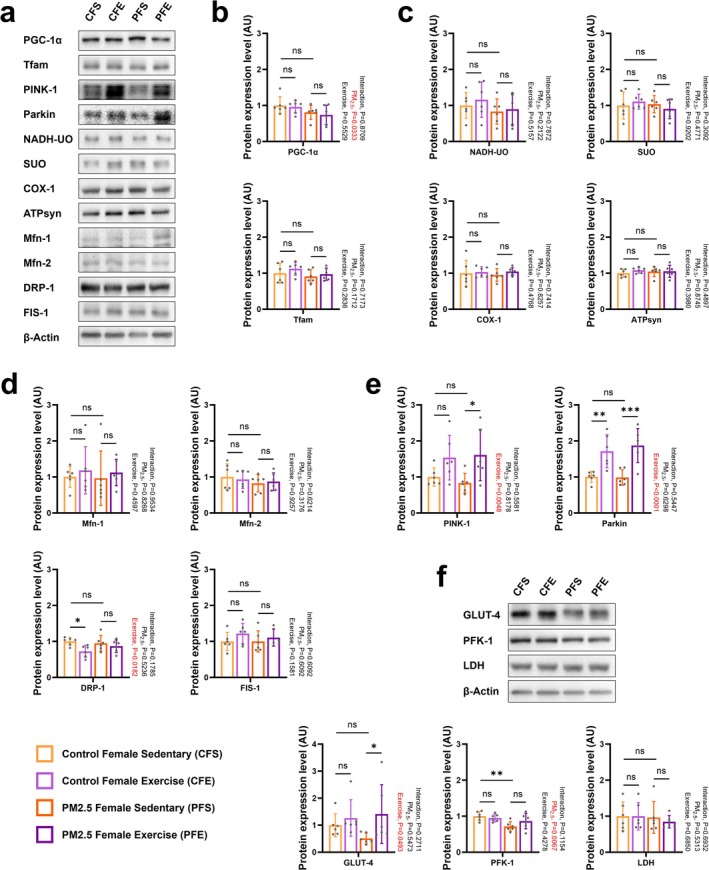
Effect of combined exercise after gestational PM_2.5_ exposure on the skeletal muscle mitochondria and glucose metabolism of female offspring. (a) Representative western blot images of PGC‐1α, Tfam, PINK‐1, Parkin, NADH‐UO, SUO, COX‐1, ATPsyn, Mfn‐1, Mfn‐2, DRP‐1, FIS‐1 and β‐actin in the gastrocnemius muscle. (b) Expression levels of PGC‐1α and Tfam in the gastrocnemius muscle. (c) Expression levels of NADH‐UO, SUO, COX‐1 and ATPsyn in the gastrocnemius muscle. (d) Expression levels of Mfn‐1, Mfn‐2, DRP‐1 and FIS‐1 in the gastrocnemius muscle. (e) Expression levels of PINK‐1 and Parkin in the gastrocnemius muscle. (f) Representative western blot images and expression levels of GLUT‐4, PFK‐1 and LDH in the gastrocnemius muscle. Every group *n* = 6. Data were presented as mean ± SD. Data were analysed by two‐way ANOVA (*p < 0.05; **p < 0.01; ***p < 0.001; ns, not significant, *p* > 0.05).

Different from male offspring, although descendants of PFS showed mitochondrial damage in their soleus muscles (Figure 7d), the problem of mitochondrial damage was significantly reduced after long‐term combined exercise intervention (Figure 7d). This was made possible by PFS offspring having normal‐functioning mitochondria, as evidenced by comparable levels of mitochondrial function–related proteins involved in biogenesis (Figure 8a,b), electron transport chain enzymes (Figure 8a,c), fusion/fission proteins (Figure 8a,d) and mitophagy proteins (Figure 8a,e) compared with CFS offspring. Mitochondrial damage in female offspring due to gestational exposure to PM_2.5_ was ameliorated by combined exercise, which enhanced the expression of the mitophagy‐related proteins PINK‐1 and Parkin (Figure 8a,e). Similarly, CFE offspring showed significantly enhanced expression of Parkin (Figure 8a,e). Similar to PMS offspring, PFS offspring showed significantly reduced expression of muscle glucose metabolism–related proteins PFK‐1 compared with CFS offspring (Figure 8f). These results suggest that the gestationally PM_2.5_‐exposed skeletal muscle system recovers to a greater extent in female than male mice over time and that combined exercise upregulates mitochondrial phagocytosis in these females.

## 4 Discussion

Skeletal muscle development directly affects motor and behavioural abilities [23]. Skeletal muscle, as the largest metabolic organ, has a direct impact on homeostasis of glucose and lipid metabolism [24] and secretes a variety of myokines that regulate homeostasis of the body's internal environment and other systems through endocrine and paracrine effects [25, 26], making it essential for maintaining quality of life and health throughout life.

A growing number of studies have shown that gestational PM_2.5_ exposure can lead to postnatal foetal low birth weight problems [27]. We observed similar outcomes in this study; however, prior studies have not explicitly addressed how foetal low birth weight impacts skeletal muscle development and function during the growth period. Our findings demonstrate that PM_2.5_‐induced low birth weight was reversed during the growth phase, with affected individuals showing accelerated growth compared with normal controls (Figure 1c). Notably, male offspring exposed to PM_2.5_ during gestation exhibited faster growth recovery and body weight normalization than females. This phenomenon likely reflects an autologous developmental compensation mechanism, characterized by catch‐up growth [28]. This process, driven by metabolic and endocrine adaptations, aims to restore normal growth patterns but may predispose individuals to long‐term metabolic risks, including insulin resistance and altered GLUT‐4 expression, as described by Xing et al. [29] and corroborated in our findings (Figure 1c; Figure 5f; Figure 8f). Future research is necessary to elucidate the underlying molecular and physiological mechanisms guiding this compensatory growth.

Skeletal muscle mitochondria were significantly smaller in offspring exposed to PM_2.5_ during embryonic development compared with normal offspring, despite the recovery in low birth weight (Figure 2a). This size discrepancy is accompanied by compensatory upregulation of mitochondrial biosynthesis and electron transport chain proteins, alongside a nearly fourfold increase in FIS‐1 expression (Figure 2c). Gestational PM_2.5_ exposure affects mitochondrial dynamics and function in skeletal muscle, as demonstrated by alterations in mitophagy and biogenesis pathways. A key regulator, PGC‐1α, plays a critical role in mitochondrial quality control, driving both mitochondrial biogenesis via NRF1 and mitophagy through the PINK1–Parkin pathway [30]. The dysregulation of these processes in PM_2.5_‐exposed offspring contributes to reduced mitochondrial biosynthesis and impaired autophagic clearance of damaged mitochondria, as observed in this study.

While mitochondrial size differences normalized by the end of the growth period, gestational PM_2.5_ exposure caused extensive and persistent mitochondrial damage in skeletal muscle, particularly in male offspring. Male mice showed decreased expression of Tfam, PINK1, Parkin, DRP‐1 and FIS‐1 (Figure 5). Interestingly, 8 weeks of combined exercise revealed sex‐specific differences in skeletal muscle response. In male normal exercise groups, P70S6K (Figure 3e), PGC‐1α (Figure 5d) and mitochondrial electron transport chain enzymes (Figure 5e) increased, alongside larger mitochondrial size. However, PME mice failed to show these exercise‐induced improvements, indicating reduced sensitivity of skeletal muscle biogenesis signalling to exercise stimulation and decreased PINK1‐mediated mitophagy.

In contrast, female normal and PFE exercise groups did not exhibit increased expression of protein synthesis factors or biogenesis regulators (Figures 6e and 8b) but showed elevated levels of PINK1 and Parkin, enhancing mitophagy flux (Figure 8e). This adaptive response effectively reduced mitochondrial damage in PFE mice (Figure 7d), supporting the critical role of mitophagy in maintaining mitochondrial health. These mechanisms highlight the disrupted mitophagy signalling in PM_2.5_‐exposed offspring as a central factor in mitochondrial damage and impaired recovery.

Despite the finding of a previous study that long‐term endurance exercise had a significant ameliorative effect on mitochondrial damage and low levels of mitochondrial biogenesis due to PM_2.5_ exposure [9], Fan et al. used long‐term PM_2.5_ exposure in conjunction with a 6‐month endurance exercise intervention, which reversed the adverse effects of chronic PM_2.5_ exposure on skeletal muscle [9]. In contrast, our study focused on short‐term exposure during critical developmental periods and assessed the effects of a combined exercise regimen. The observed differences in modulatory effects are likely attributable to variations in intervention models and exercise programme parameters, such as duration, intensity, frequency and timing. For instance, Liu et al. (2024) demonstrated that 12 weeks of endurance training enhanced mitochondrial oxidative metabolism and reduced PM_2.5_‐induced damage in skeletal muscle, emphasizing the critical role of programme duration in eliciting protective adaptations. Additionally, short‐term interventions during critical developmental windows, when organisms are more susceptible to oxidative stress and mitochondrial dysfunction, may require prolonged or higher intensity exercise programmes to achieve comparable benefits [5].

Furthermore, exercise intensity and mode play crucial roles. Moderate‐intensity aerobic exercise has been shown to better regulate oxidative stress and improve mitochondrial function compared with low‐intensity or short‐duration training. Jin et al. highlighted that such training effectively mitigated systemic oxidative damage induced by air pollution. These findings underline the importance of tailoring exercise programmes to specific exposure scenarios and biological contexts [31]. To develop more effective exercise regimens for mitigating PM_2.5_‐induced skeletal muscle damage, future studies should explore diverse exercise modalities, intensities and frequencies while considering the timing of both exposure and intervention. This comprehensive approach may provide a more robust framework for countering the adverse effects of air pollution on musculoskeletal health.

Our experiments showed that 8 weeks of combined exercise had different effects in males and females. However, there are few sex‐related validations of exercise effects. A number of prior studies have demonstrated that differences in oestrogen levels directly affect antioxidant function [32], mitochondrial function [33], immune function [34] and skeletal muscle homeostasis [35]. Thus, the differences in exercise effects seen in our study may be due to the variability in oestrogen levels between sexes. It is also important to consider the role of sex hormones, including testosterone, in muscle mass and function. PM_2.5_ exposure may disrupt the secretion of these hormones, particularly testosterone in males, which plays a key role in muscle maintenance. Testosterone influences skeletal muscle mass, and alterations in its levels due to environmental stressors like PM_2.5_ could explain some of the sex‐based differences observed in skeletal muscle outcomes.

PM_2.5_ exposure has been shown to affect hormone levels through mechanisms like oxidative stress, which may influence the hypothalamic–pituitary–gonadal (HPG) axis. Disruption of this axis has been linked to decreased testosterone production, as seen in studies on mice exposed to concentrated ambient PM_2.5_, where a reduction in sperm quality and testosterone biosynthesis was observed [36, 37]. Environmental pollutants, including air pollution, induce oxidative stress, which has been associated with conditions like hypogonadism and reduced testosterone production, potentially contributing to the impaired muscle development seen in male offspring exposed to PM_2.5_. Similarly, PM_2.5_ exposure may also influence oestrogen levels through oxidative stress‐induced damage to oestrogen receptor signalling pathways, disrupting normal endocrine functions and affecting reproductive health in both sexes. These findings suggest that PM_2.5_ exposure acts as an endocrine disruptor, particularly affecting reproductive hormones like testosterone and oestrogen, with long‐term consequences for skeletal muscle health and fertility. However, elucidating the exact mechanism behind these hormonal disruptions, especially their effects on muscle outcomes, remains an important area for future investigation.

Although the mechanism by which maternal PM_2.5_ exposure affects the mitochondrial function of skeletal muscle in offspring is unclear, direct exposure to PM_2.5_ and genetic exposure both show similar mitochondrial‐specific effects. In previous studies, direct exposure to PM_2.5_ has been shown to cause long‐term damage to skeletal muscle mitochondria and impaired dynamics regulation, with the damage effect being more pronounced in women [38]. Interestingly, mitochondrial genes are more influenced by maternal inheritance, and maternal ageing and mitochondrial‐related diseases can directly cause mitochondrial dysfunction in offspring [39, 40]. This seems to be a good explanation for the mitochondrial specificity of the genetic damage effect in offspring. In future research, it is necessary to clarify the maternal influence pathway in the effect of PM_2.5_ genetic exposure.

This study demonstrates that gestational PM_2.5_ exposure impairs skeletal muscle development, induces mitochondrial damage and reduces exercise capacity in offspring. Combined exercise had bidirectional, sex‐specific effects: male offspring exhibited reduced responsiveness to exercise, with persistent mitochondrial damage, whereas female offspring showed improved mitochondrial health through increased mitophagy flux. The findings underscore the importance of sex differences and the need to optimize exercise regimens to mitigate air pollution‐induced damage during critical developmental periods. Future research should explore the molecular mechanisms underlying these adaptations and investigate diverse exercise modalities and intensities to develop effective interventions.

## Author Contributions

**Zilin Wang:** writing – review and editing, writing – original draft, validation, formal analysis, visualization. **Wenduo Liu:** writing – review and editing, writing – original draft, validation, visualization, data curation. **Hyun‐Jaung Sim:** validation, supervision, funding acquisition. **Jeong‐Chae Lee:** resources, funding acquisition. **Sung‐Ho Kook:** writing – review and editing, writing – original draft, conceptualization, resources, methodology, funding acquisition. **Sang Hyun Kim:** writing – review and editing, conceptualization, resources, validation, project administration, methodology, funding acquisition.

## Conflicts of Interest

The authors declare no conflicts of interest.

## Supporting information

Supporting information.
**Figure S1:** Effect of gestational PM2.5 exposure on exercise capacity and fat mass of offspring 4 weeks of age. (a) Endurance capacity. (b) Grip strength test. (c) Epididymal (or Ovarian) & retroperitoneal fat pad masses. Every group n=4; Data are presented as mean ± SD. Data were analyzed by two‐way ANOVA (*p < 0.05; **p < 0.01; ***p < 0.001; ns, not significant, *p* > 0.05).
**Figure S2:** Schematic experimental design of the combined exercise program.
**Table S1:** List of the chemical compositions, formula, and dry mass fractions of organic and inorganic species used in this study.
**Table S2:** Mean PM concentration in the exposure chamber.
