# Supplementary material for: Effects of Combined Exercise Training on Modulating Fine Particulate Matter–Induced Skeletal Muscle Damage in Offspring Gestationally Exposed
Source: J Cachexia Sarcopenia Muscle. 2025 Sep 10;16(5):e70047. doi: 10.1002/jcsm.70047 (PMC12421413; doi:10.1002/jcsm.70047)
Supplement: Supplementary file 1 — Supporting information. Figure S1: Effect of gestational PM2.5 exposure on exercise capacity and fat mass of offspring 4 weeks of age. (a) Endurance capacity. (b) Grip strength test. (c) Epididymal (or Ovarian) & retroperitoneal fat pad masses. Every group n=4; Data are presented as mean ± SD. Data were analyzed by two‐way ANOVA (*p < 0.05; **p < 0.01; ***p < 0.001; ns, not significant, p > 0.05). Figure S2: Schematic experimental design of the combined exercise program. Table S1: List of the chemical compositions, formula, and dry mass fractions of organic and inorganic species used in this study. Table S2: Mean PM concentration in the exposure chamber. [file JCSM-16-e70047-s001.docx]

**Effects of combined exercise training on modulating fine particulate matter-induced skeletal muscle damage in offspring gestationally exposed**

Zilin Wang^1#^, Wenduo Liu^1#^, Hyun-Jaung Sim^2,3^, Jeong-Chae Lee^2,3^, Sung-Ho Kook^3^*, Sang Hyun Kim^1^*

^1^Department of Sports Science, College of Natural Science, Jeonbuk National University, Jeonju 54896, Republic of Korea; ^2^Cluster for Craniofacial Development and Regeneration Research, Institute of Oral Biosciences and School of Dentistry, Jeonbuk National University, Jeonju 54896, Korea; ^3^Department of Bioactive Material Sciences, Research Center of Bioactive Materials, Jeonbuk National University, Jeonju 54896, Republic of Korea.

^*^Address correspondence to: Sung-Ho Kook, Department of Bioactive Material Sciences, Jeonbuk National University, Jeonju 54896, Republic of Korea, Phone: +82-63-270-3327, Fax: +82-63-270-4312, E-mail: kooksh@jbnu.ac.kr

Sang Hyun Kim, Department of Sports Science, College of Natural Science, Jeonbuk National University, Jeonju 54896, Republic of Korea, Phone: +82-63-270-2853, Fax: +82-63-270-4234, E-mail: [sh5275@jbnu.ac.kr](mailto:sh5275@jbnu.ac.kr)

^#^ These authors contributed equally to this work.

**Supplementary Figure**

**Supplementary Figure 1. Effect of gestational PM_2.5_ exposure on exercise capacity and fat mass of offspring 4 weeks of age.** (a) Endurance capacity. (b) Grip strength test. (c) Epididymal (or Ovarian) & retroperitoneal fat pad masses. Every group n=4; Data are presented as mean ± SD. Data were analyzed by two-way ANOVA (* p < 0.05; ** p < 0.01; *** p < 0.001; ns, not significant, p > 0.05).

Supplementary Figure 2. Schematic experimental design of the combined exercise program.

**Supplementary Figure 1**

**
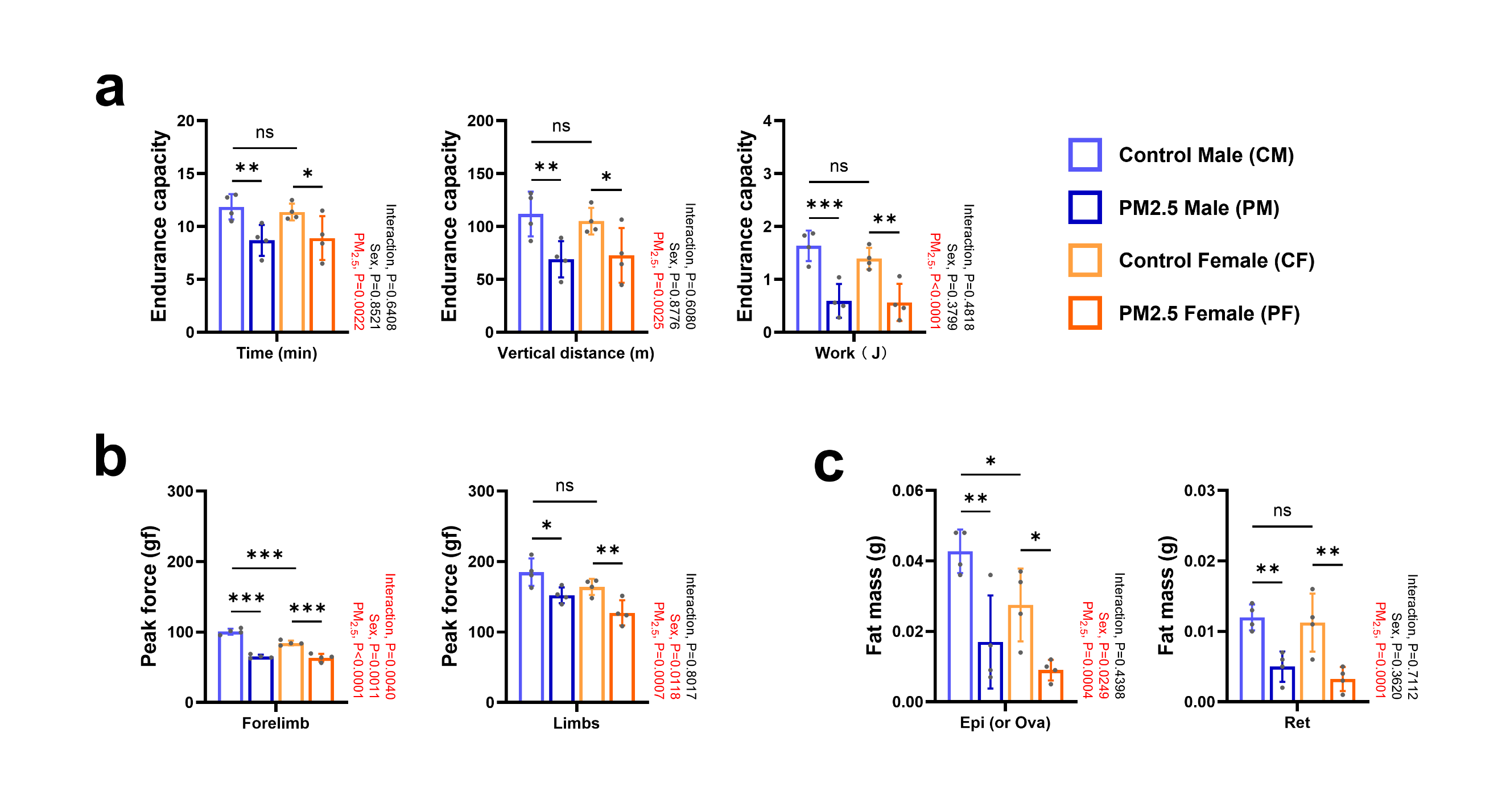
**

**Supplementary Figure 2**

**
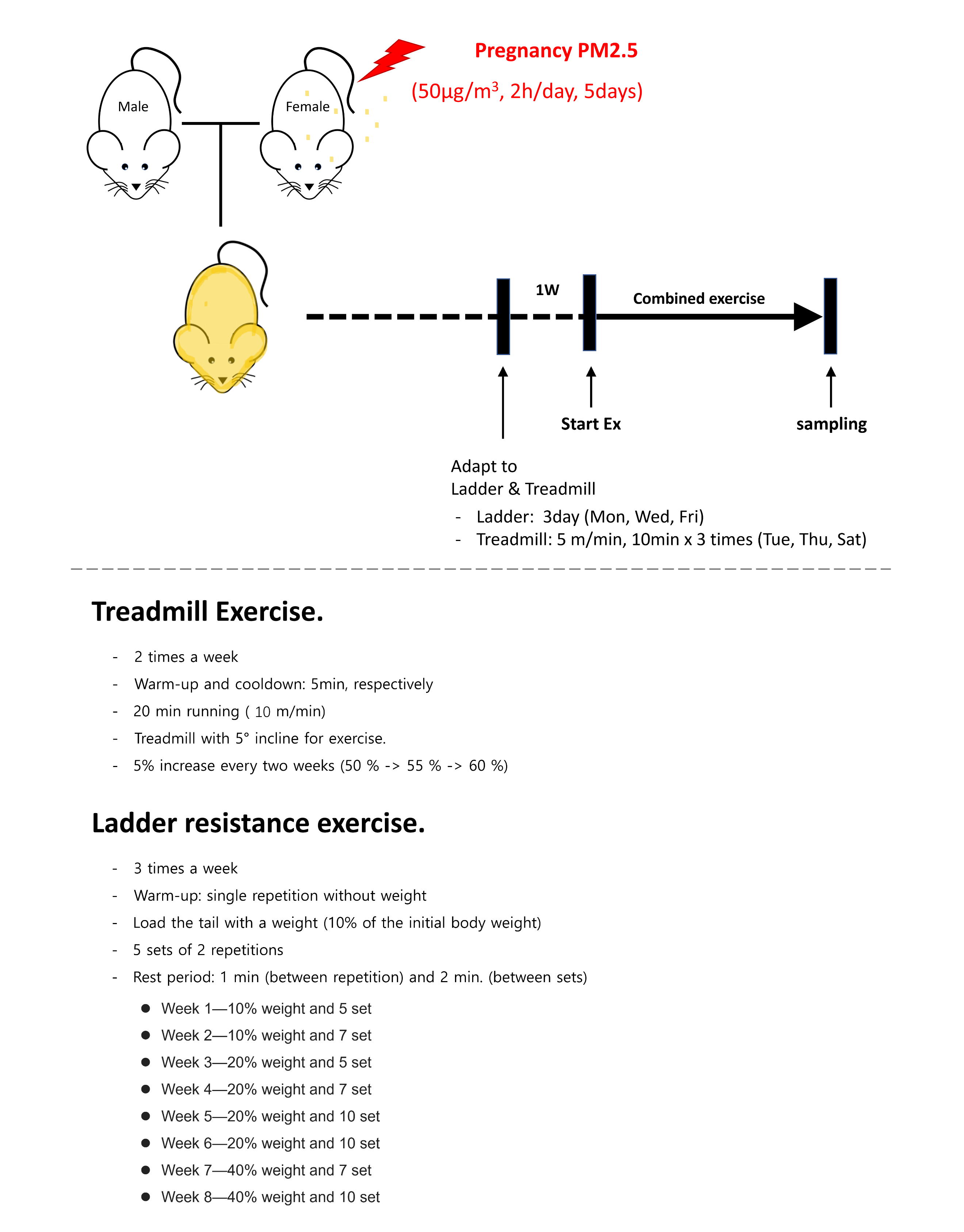
**

**Supplementary Table**

**Supplementary Table 1.** List of the chemical compositions, formula, and dry mass fractions of organic and inorganic species used in this study.

| **Functional Group** | **Components** | **Formula** | **Density (g/cm^3^) at 295 K^*^** | **Dry mass fraction (%)** |
| --- | --- | --- | --- | --- |
| Monocarboxylic acid | Acetate | C_2_H_3_O_2_ | 1.05 | 6.25 |
| Dicarboxylic acid | Oxalic acid | C_2_H_2_O_4_ | 1.90 | 6.25 |
|  | Malonic acid | C_3_H_4_O_4_ | 1.62 | 6.25 |
|  | Glutaric acid | C_5_H_8_O_4_ | 1.35 | 6.25 |
| Polyols | Glycerol | C_3_H_8_O_3_ | 1.26 | 6.25 |
| Sugars | Sucrose | C_12_H_22_O_11_ | 1.59 | 6.25 |
| Aromatics | 2,5-Dihydroxybenzoic acid | C_7_H_6_O_3_ | 1.55 | 6.25 |
| Amino acid | Glycine | C_2_H_5_O_2_N | 1.61 | 6.25 |
| Inorganic salts | Ammonium sulfate | (NH_4_)_2_SO_4_ | 1.77 | 25 |
|  | Ammonium nitrate | NH_4_NO_3_ | 1.72 | 25 |

^*^ Values of measured densities are from www.chemicalbook.com

**Supplementary Table 2.** Mean PM concentration in the exposure chamber.

| Method | Particle counter (μg/m^-3^) |
| --- | --- |
| Batch1 | 55.3 ± 7.2 |
| Batch2 | 46.6 ± 5.8 |
| Batch3 | 52.8 ± 11.7 |
| Batch4 | 50.8 ± 8.8 |
| Batch5 | 53.4 ± 13.4 |

**Supplementary References**

1. S. Gilardoni, S. Liu, S. Takahama, et al., “Characterization of organic ambient aerosol during MIRAGE 2006 on three platforms,” *Atmos. Chem. Phys.* 9 (2009): 5417–5432.
2. L. N. Hawkins, L. M. Russell, D. S. Covert, P. K. Quinn and T. S. Bates, “Carboxylic acids, sulfates, and organosulfates in processed continental organic aerosol over the Southeast Pacific Ocean during VOCALS-REx 2008,” *J. Geophys. Res. Atmos.* 115 (2010): D13.
3. T. Y. Ling and C. K. Chan, “Partial crystallization and deliquescence of particles containing ammonium sulfate and dicarboxylic acids,” *J. Geophys. Res. Atmos.* 113 (2008): D14.
4. G. Bhattarai, J. B. Lee, M. H. Kim, et al., “Maternal exposure to fine particulate matter during pregnancy induces progressive senescence of hematopoietic stem cells under preferential impairment of the bone marrow microenvironment and aids development of myeloproliferative disease,” *Leukemia* 34 (2020): 1481-1484.
5. V. S. Massicotte, N. Frara, M. Y. Harris, et al., “Prolonged performance of a high repetition low force task induces bone adaptation in young adult rats, but loss in mature rats,” *Exp. Gerontol.* 72 (2015): 204–217.
